# In Vitro Evaluation of Multifocal Intraocular Lenses Based on the Point Spread Function: Optical Performance and Halo Formation

**DOI:** 10.3390/jcm14238368

**Published:** 2025-11-25

**Authors:** Anabel Martínez-Espert, Salvador García-Delpech, Walter D. Furlan

**Affiliations:** 1 Departamento de Óptica, Optometría y CC de la Visión, Universitat de València, 46100 Burjassot, Spain; 2 Clínica Aiken, Fundación Aiken, 46004 Valencia, Spain

**Keywords:** multifocal intraocular lens, chromatic aberration, optical bench, halos

## Abstract

**Background**: Trifocal and extended depth-of-focus (EDoF) multifocal intraocular lenses (MIOLs) are currently widely used after cataract surgery to restore vision at multiple distances. In vitro studies of MIOLs are necessary to evaluate their optical behavior providing surgeons with evidence to support the appropriate selection of the best lens for each patient. **Methods**: The FineVision POD F, Acriva Trinova Pro C, AT LARA 829MP, and AcrySof IQ Vivity lenses were assessed using a dedicated optical bench. Optical quality was quantified using the through-focus modulation transfer function (TF-MTF) and the area under the modulation transfer function (MTFa), both calculated from the point spread function (PSF). Halo formation was qualitatively analyzed. **Results**: The FineVision POD F and Acriva Trinova Pro C lenses exhibited trifocal behavior, with optical performance varying according to pupil size and wavelength. The AT LARA 829MP lens functioned as a low-addition bifocal under monochromatic green light but demonstrated EDoF characteristics under polychromatic illumination. The AcrySof IQ Vivity lens displayed an EDoF profile derived from the superposition of multiple closely spaced foci under polychromatic evaluation. Halo assessment revealed lens-dependent differences, with the AcrySof IQ Vivity showing the smallest halo extent. **Conclusions**: This in vitro study demonstrates differences in the optical and chromatic performance of trifocal and EDoF IOLa. Trifocal designs showed variable behavior related to diffraction orders the use but generally favored far vision under mesopic conditions, with similar trends observed in EDoF lenses. EDoF designs produced fewer halos than trifocals. These quantitative findings may translate into clinically relevant effects, supporting MIOL selection tailored to patient needs and improving the predictability and personalization of surgical outcomes toward greater spectacle independence.

## 1. Introduction

Multifocal intraocular lenses (MIOLs) have evolved significantly over the past decades, aiming to restore vision at different distances after cataract surgery and to reduce dependence on optical aids such as eyeglasses or multifocal contact lenses [1]. Among them, trifocal MIOLs and extended depth-of-focus (EDoF) intraocular lenses represent two of the most widely adopted approaches [2].

The optical design of MIOL (whether based on diffractive or refractive principles) inevitably results in the simultaneous projection of both in-focus and out-of-focus images onto the retina. This overlap reduces retinal contrast and can lead to dysphotopsias, with halos being among the most prominent and frequently reported visual phenomena in patients implanted with MIOLs [3,4].

Trifocal lenses have demonstrated effective improvement in distance, intermediate, and near vision, providing a broad visual range that results in a high degree of spectacle independence. In contrast, while EDoF lenses are often reported to extend the depth of focus and to induce fewer dysphotopsias than trifocal lenses [1,2], the evidence for some designs remains inconsistent, and patient-reported outcomes vary considerably [5]. These uncertainties highlight the need for objective comparisons under controlled conditions.

The in vitro evaluation of MIOLs using specific optical merit functions, such as the point spread function (PSF) and the modulation transfer function (MTF), provides objective insights into the optical performance of each design. This approach enables a deeper understanding of their image formation behavior, their pupil size dependence, and their tendency to generate halos. Consequently, it becomes possible to identify the strengths and limitations of each design and to provide valuable information that supports ophthalmologists in making personalized selections based on the lifestyles and visual expectations of the patient. Moreover, these metrics are particularly useful for predicting visual acuity across different vergences, thereby allowing a more accurate assessment of the potential impact on visual quality after implantation.

The ISO 11979 standard [6] regulates the optical properties and test methods that must be evaluated for intraocular lenses. Several commercial instruments have been designed to comply with this standard; however, the results reported for the same MIOL using two different instruments do not always coincide [7,8], likely due to differences in their underlying optical principles. Furthermore, most of these instruments do not include clinically relevant parameters such as the area under the modulation transfer function (MTFa) or assessments performed under polychromatic illumination.

To overcome these limitations, we implemented an optical bench in accordance with the aforementioned standard [6], which also provides greater flexibility in terms of illumination conditions (multiple wavelengths and white light) and measurable optical parameters (PSF, MTF, and MTFa) [9,10].

Taking into account all of the aforementioned considerations, the aim of this study was to provide clinicians with new evidence regarding the optical properties of four commercially available MIOLs with distinct optical designs: two trifocal lenses (the FineVision POD F, PhysIOL, Liège, Belgium, and the Acriva Trinova Pro C, VSY Biotechnology GmbH, Leinfelden-Echterdingen, Germany) and two EDoF lenses (the AT LARA 829MP, Carl Zeiss Meditec, Jena, Germany, and the AcrySof IQ Vivity, Alcon Laboratories, Fort Worth, TX, USA). These lenses were evaluated and compared for the first time using the same optical setup under polychromatic illumination.

## 2. Materials and Methods

### 2.1. MIOLs Description

The FineVision POD F lens (PhysIOL, Liège, Belgium) is a diffractive trifocal MIOL with a kinoform profile. Its anterior surface consists of two superimposed apodized diffractive profiles. One of these profiles uses the *m* = 0 and *m =* +1 diffraction orders to distribute energy between the distance and intermediate foci, while the other one uses the orders *m*′ = 0 and *m*′ = +1 to distribute energy between the far and near foci. In this way, the lens provides an intermediate addition of +1.75 D and a near addition of +3.50 D. Its posterior surface is aspheric and designed to induce a spherical aberration (SA) of –0.11 μm [11]. This MIOL was the first trifocal lens on the market [11] and is a well-established multifocal reference lens with a long-standing clinical track record.

The Acriva Trinova Pro C lens (VSY Biotechnology GmbH, Leinfelden-Echterdingen, Germany) is a diffractive trifocal MIOL with an apodized sinusoidal profile on its anterior surface. In this case, the diffractive profile provides three diffraction orders: *m* = −1 for distant vision, *m* = 0 for intermediate vision, and *m* = +1 for near vision. It has nominal additions of +1.80 D for intermediate vision and +3.60 D for near vision and its posterior surface is aspheric and designed to induce a SA of –0.1 μm [12,13]. According to the manufacturer, its sinusoidal profile minimizes the incidence of dysphotopsias. Furthermore, they claim that the “adaptive technology” dynamically adjusts light distribution according to lighting conditions, promoting continuous, stable, and comfortable vision across various illumination environments [14].

The AT LARA 829MP lens (Carl Zeiss Meditec, Jena, Germany) is an EDoF diffractive lens that incorporates the technology registered by the manufacturer under the name “smooth microphase” [8]. This technology features a diffractive surface with much shallower angles that can be manufactured more precisely, thus minimizing the amount of light scatter [15]. It uses the *m* = +1 diffraction order to generate far focus and the *m* = +2 diffraction order to generate intermediate focus, achieving an EDoF effect as a low-addition bifocal lens at its design wavelength. It is a neutral SA lens due to its aspheric posterior surface.

The AcrySof IQ Vivity lens (Alcon Laboratories, Fort Worth, TX, USA) uses the manufacturer-defined “X-WAVE” technology on its anterior surface to stretch and shift the wavefront, thereby extending the range of vision from distance to intermediate. Its design features a 1 μm high annular elevation within the central 2.2 mm of the optical zone [16,17]. This surface is also aspheric and is designed to induce negative SA [18]. According to the manufacturer, this design contributes to reduced incidence of halos and glare compared to traditional diffractive presbyopia correcting IOLs.

Table 1 summarizes the technical specifications of the evaluated MIOLs.

### 2.2. Experimental Setup

A custom-made optical bench, conceived to accomplish the requirements of the International Organization for Standardization ISO 11979-2, 2014 [6], was employed in this study. The optical system, described in detail elsewhere [9,10]. This experimental setup was improved to obtain, automatically in a single measurement, the values of the axial PSF, from which the MTF and the MTFa of MIOLs can be obtained [19]. Let us recall that, in general terms, the PSF represents the image of a point-like object produced by a given optical system. The PSF determines the intensity distribution within an image, as it describes how light from a point source is spread over a finite area. It is a three-dimensional function that provides a complete characterization of the imaging properties of the system. In the case of a MIOL, the PSF serves as the fundamental, or “mother,” function from which other optical performance metrics, such as the MTF and the MTFa curve, can be derived. The PSF of a MIOL is influenced by several parameters, including the pupil diameter, the wavelength of the incident light, and the refractive index of the MIOL material. Consequently, a PSF with higher values indicates superior optical performance of the MIOL in terms of image sharpness and overall visual quality.

**Figure 1:**
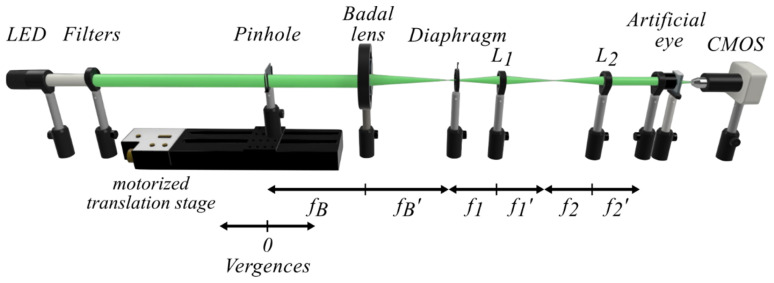
The system automatically records the PSF generated by an artificial eye, using a 30-μm pinhole as the object, which is axially displaced to simulate different object vergences (see the main text for details).

Figure 1 shows the experimental setup. The point-like object, a 30 μm pinhole, was illuminated by a collimated beam from a cold white LED (Thorlabs MCWHL5, Newton, NJ, USA), which was sequentially filtered with different chromatic filters (10 nm bandwidth) centered at λ = 450 nm λ = 500 nm, λ = 550 nm, 600 nm, and λ = 650 nm (Thorlabs FB450-10, FB550-10, FB600-10, FB650-10, respectively). The pinhole object was moved axially with a motorized translation stage (Thorlabs LTS300/M, 300 mm travel range, ±5 μm accuracy) to generate vergences ranging from −1 to +5 D in steps of 0.1 D. Vergences were measured from the object focal plane of the Badal lens of focal length 160 mm (see Figure 1). As the cornea of the artificial eye was placed at the image focal plane of the Badal lens; the angular size of the pinhole images was independent of the pinhole position; so that, IOLs with different powers do not produce variations in the final image magnification; like in a focimeter [20]; and therefore, PSFs and MTFs of different MIOL designs and power can be directly compared. The artificial eye consisted of an achromatic lens with nearly zero spherical aberration (Melles Griot LA034 27.8D) (ISO 11979-2:2014 Model 1 cornea [6]) which was selected to avoid the mutual interaction between LCA and spherical aberration of the different MIOL’s models [9]. The MIOLs under test were placed in a wet cell filled with a saline solution. A variable mechanical diaphragm, acting as the artificial pupil of the eye, was projected onto the anterior surface of the MIOL. Images provided by the artificial eye were captured with a CMOS sensor (EO-5012C, Edmund Optics, Barrington, NJ, USA) coupled to a 5× microscope objective.

The study was performed with pupil diameters of 3.0 mm and 4.5 mm to emulate photopic and mesopic conditions [21,22,23], respectively. The PSFs obtained at five wavelengths were weighted according to the photopic spectral luminous efficiency function V(λ) (CIE 1924) [24] with the following weights: 0.0380 (450 nm), 0.3230 (500 nm), 0.9950 (550 nm), 0.6310 (600 nm), and 0.1070 (650 nm).

To isolate the polychromatic optical performance of the MIOLs from residual chromatic aberration introduced by the optical elements, the system was calibrated without a MIOL by measuring the longitudinal chromatic aberration (LCA), which was later compensated numerically. The LCA at the principal foci of each lens was calculated as the difference between the focal planes of two extreme wavelengths (λ = 450 nm and λ = 650 nm).

Then, the PSF images were processed in LabView to calculate the MTFs over defined spatial frequencies and vergences using the Fourier Transform (FT) of the PSF according to Equation (1):

(1) $$MTF\left(f_{x},f_{y}\right)\;=\;\left|FT\;\left\{PSF\left(x_{i},y_{i}\right)\right\}\right|$$

From these MTFs, both the TF-MTF and MTFa were calculated. The TF-MTF was obtained with λ = 550 nm at a spatial frequency of 50 line pairs per millimeter (lp/mm). The MTFa values between 0 lp/mm and 50 lp/mm were calculated for the three monochromatic wavelengths and for polychromatic light using the V(λ) function.

Finally, halo formation under monochromatic and mesopic conditions was qualitatively assessed. A 150 μm pinhole and the λ = 550 nm chromatic filter were used with a pupil diameter of 4.5 mm. Images were captured at the main foci of each lens.

## 3. Results

**Figure 2:**
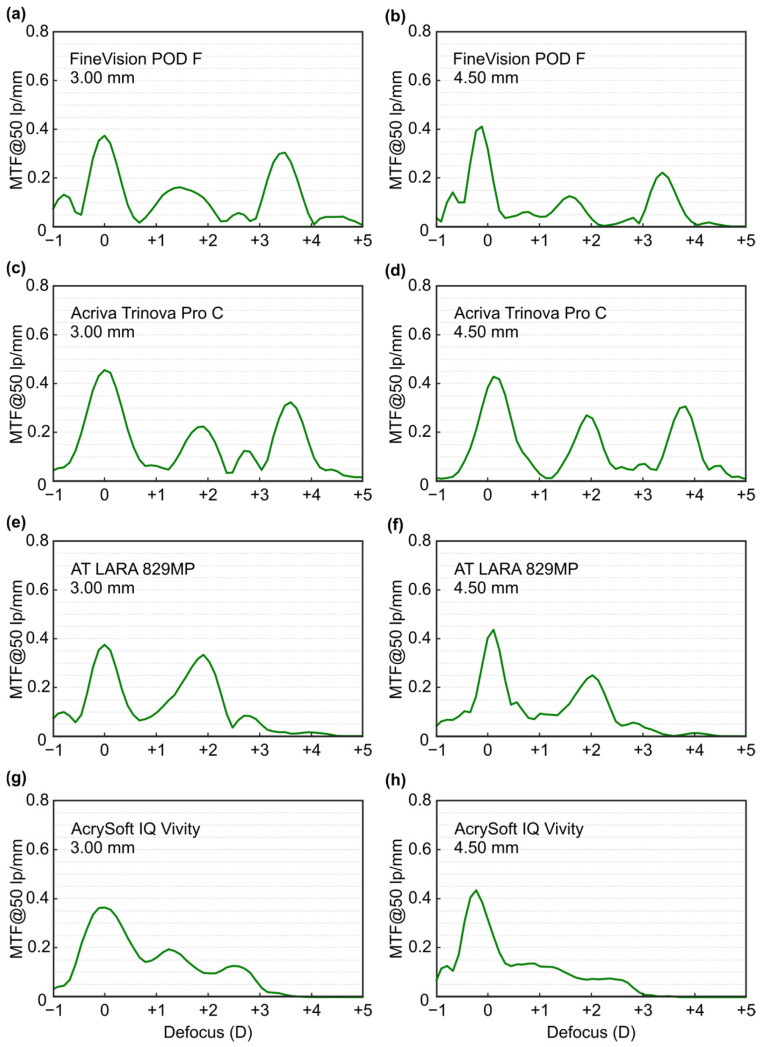
Experimental Through-Focus Modulation Transfer Function (TF-MTF) curves ob-tained for each multifocal intraocular lens at a spatial frequency of 50 lp/mm with pu-pil apertures of 3.0 mm and 4.5 mm: (**a**) FineVision POD F, 3.0 mm pupil; (**b**) FineVision POD F, 4.5 mm pupil; (**c**) Acriva Trinova Pro C, 3.0 mm pupil; (**d**) Acriva Trinova Pro C, 4.5 mm pupil; (**e**) AT LARA 829MP, 3.0 mm pupil; (**f**) AT LARA 829MP, 4.5 mm pu-pil; (**g**) AcrySof IQ Vivity, 3.0 mm pupil; (**h**) AcrySof IQ Vivity, 4.5 mm pupil.

Figure 2 shows the TF-MTF results at 50 lp/mm for the four evaluated MIOLs, values at the main foci of each lens are presented in Table 2. Both the FineVision POD F lens (Figure 2a,b) and the Acriva Trinova Pro C lens (Figure 2c,d) exhibited trifocal behavior with both pupil sizes, showing a higher MTF value at the distance focus and lower at the intermediate focus. The AT LARA lens (Figure 2e,f) provides a bifocal profile for both pupil sizes, and the MTF was higher at far focus. The AcrySof IQ Vivity lens (Figure 2g,h) exhibits a smooth transition from far to near vergences. With the larger pupil diameter, the optical performance improves for distance vision but decreases across the remaining vergence range.

**Figure 3:**
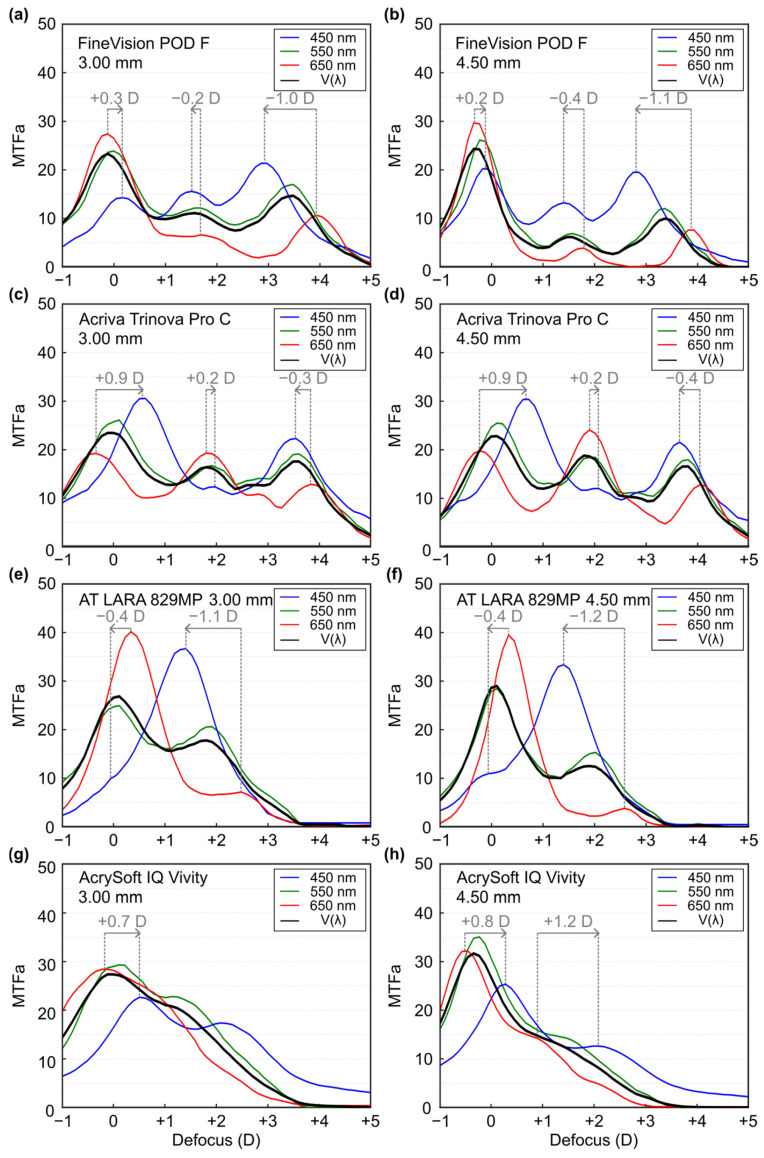
Experimental area under the Modulation Transfer Function (MTFa) graphs obtained for each multifocal intraocular lens evaluated for the spatial frequenccies between 0 lp/mm and 50 lp/mm, and for the pupil apertures of 3.0 mm and 4.5 mm: (**a**) FineVision POD F, 3.0 mm pupil; (**b**) FineVision POD F, 4.5 mm pupil; (**c**) Acriva Trinova Pro C, 3.0 mm pupil; (**d**) Acriva Trinova Pro C, 4.5 mm pupil; (**e**) AT LARA 829MP, 3.0 mm pupil; (**f**) AT LARA 829MP, 4.5 mm pupil; (**g**) AcrySof IQ Vivity, 3.0 mm pupil; (**h**) AcrySof IQ Vivity, 4.5 mm pupil. The arrow direction indicates the sign of the LCA.

Figure 3 shows the MTFa results, from 0 lp/mm to 50 lp/mm, for the three wavelengths and for the polychromatic condition. The figure also shows the experimental LCA values. Table 3 shows the MTFa values at the main foci for λ = 550 nm of each lens. Table 4 shows the experimental LCA values at the main foci of each lens.

For the FineVision POD F lens (Figure 3a,b), refractive LCA was observed at the far focus, whereas diffractive LCA (opposite in sign to the refractive LCA) was present at the intermediate and near foci for both pupil sizes. Among the three foci, the absolute magnitude of LCA was lowest at the intermediate focus and highest at the near focus (see Table 4).

For the Acriva Trinova Pro C lens (Figure 3c,d), LCA of the opposite sign was observed at the far focus compared with the intermediate and near foci. In terms of absolute magnitude, the intermediate focus showed the smallest LCA, while the largest was recorded at the far focus (see Table 4).

For the AT LARA lens (Figure 3e,f), the MTFa curves differed clearly among the three wavelengths. For the green wavelength (λ = 550 nm), the low-addition bifocal pattern was preserved. For red and blue wavelength, the curves show a clear single focus at +0.3 D and +1.4 D, respectively.

The optical performance of the AcrySof IQ Vivity lens for the green wavelength exhibited an EDoF profile (Figure 3g,h). With the larger pupil diameter, MTFa values increased at the far focus but decreased at the intermediate range (see Table 3). Regarding the chromatic behavior of the red and blue wavelengths, curves showed similar profiles to the green curve but were axially shifted. The order in which the foci appear indicates that the dominant LCA in this lens is due to refraction.

**Figure 4:**
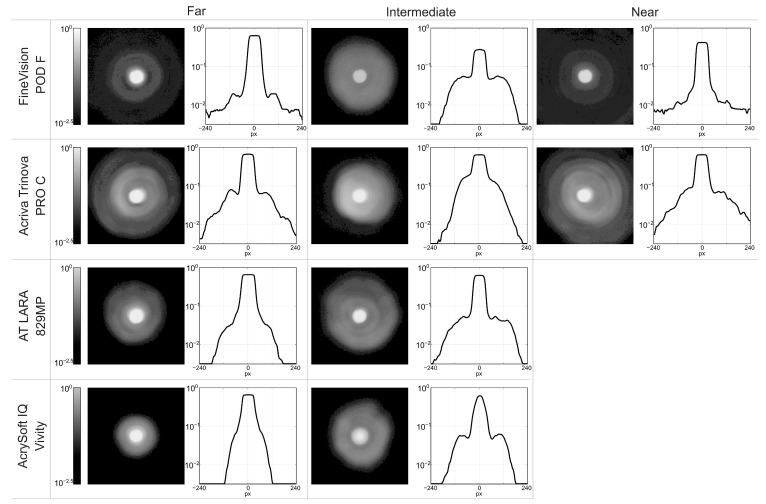
Experimental pinhole images at the main foci of each lens, for λ = 550 nm and a pupil diameter of 4.5 mm and their intensity profiles.

In addition to the TF-MTF and MTFa curves, an evaluation of halos was performed for a 4.5 mm pupil at the main foci of each lens. A logarithmic filter was then applied to the images to enhance the visualization of halos. Figure 4 shows the experimental images obtained. Table 5 shows the experimental peak intensity values of pinhole images measured at the main focus of each lens.

The FineVision POD F lens showed a smaller halo effect at the far focus and a larger effect at the intermediate focus. In the Acriva Trinova Pro C lens, halos were more uniformly distributed across the three foci, with slightly less extension at the intermediate focus. Among the EDoF designs, the AT LARA lens produced larger halos than the Vivity lens, with halos being more extensive at the intermediate focus than at the far focus in both cases. The Vivity lens showed the smallest halo and more intense image at the far focus.

## 4. Discussion

In current clinical practice, there is broad consensus among ophthalmologists that selecting the most appropriate MIOL for cataract and presbyopia correction requires a careful assessment of which model best aligns with each patient’s visual demands. To this end, independent in vitro studies evaluating the main characteristics of different models are particularly valuable, as they provide relevant information that is not disclosed by manufacturers, whose reports are often skewed by commercial interests. Within this context, data regarding chromatic responses and the expected values of visual acuity (derived from the MTFa) are especially pertinent. Another critical factor is the perception of halos associated with each MIOL design, which remains one of the most frequently reported visual disturbances among implanted patients. Within this framework, in this study we conducted a comprehensive comparative analysis of four premium commercial MIOLs with well-differentiated designs and optical properties, which (to the best of our knowledge) had not previously been evaluated on an optical bench under identical experimental conditions. We provide quantitative criteria in terms of MTFa and chromatic aberration that could help ophthalmologists to select the most appropriate design according to each patient’s visual needs—desired visual acuity values at multiple distances and tolerance to dysphotopsias—thereby improving the predictability and personalization of surgical outcomes.

Our findings clearly demonstrate that different designs, both trifocal and EDoF, yield distinct optical outcomes. In particular, as expected, we found that trifocal IOLs provide a greater range of vergences with three well-defined focal points for far, intermediate, and near vision. However, valleys occur between these foci, corresponding to regions of reduced visual quality. In addition, trifocal lenses exhibit a higher extension of halos compared to EDoF models. EDoF lenses, in turn, as were specifically developed to address these limitations, reduce the valleys in vision between focal points, potentially enhance visual comfort. Nevertheless, their main drawback is that they offer a somewhat more limited range of vergences, with reduced sharpness in near vision. These can be summarized as follows. The FineVision POD F lens exhibited consistent trifocal behavior across both pupil sizes, with higher optical performance at the far focus and reduced performance at the intermediate focus (Figure 2a,b). Under small pupil conditions (3.0 mm), higher MTF values were observed at the intermediate and near foci compared with the large pupil (4.5 mm). These results are consistent with those reported by Gatinel & Houbrechts [25] and Ruiz-Alcocer et al. [26]. MTFa measurements obtained for this lens at different wavelengths, for both pupil sizes (Figure 3a,b), revealed LCA of opposite sign at the far versus the intermediate and near foci (Table 4), reflecting the diffractive design of the addition. These results are in agreement with those published by Loicq et al. [27]. Halo evaluation of this MIOL (Figure 4) demonstrated that the halo at the far focus was dimmer and less extended than those observed at the intermediate and near foci. However, at the intermediate focus, performance was poorest, with a larger halo and lower central intensity.

The TF-MTF for the Acriva Trinova Pro C lens exhibits trifocal behavior under monochromatic light (λ = 550 nm) (Figure 2c,d). In this lens the larger pupil diameter improves intermediate vision. This is a characteristic feature of lenses with sinusoidal diffractive profiles and is consistent with recent reports by several authors [13,28]. However, the MTFa defocus curves for different wavelengths do not follow the same trend; under blue light, the behavior resembled that of a bifocal lens, and the intermediate diffractive focus showed the lowest chromatic aberration (Figure 3c,d). This is also a characteristic of lenses with sinusoidal diffractive profiles. Łabuz et al. [7] reported similar results, although the artificial eye of the commercial device used in their study incorporated a different corneal model, which induced positive SA and produced axial shifts in the blue and red foci. Halo analysis of this lens (Figure 4) demonstrated an approximately uniform distribution across the three foci, with the intermediate focus exhibiting a slightly smaller halo. These results are comparable to those previously reported by Vega et al. [29], for the Acriva Trinova lens (which shares the same profile as the Acriva Trinova Pro C but is not apodized) who also observed a balanced distribution among the three foci under monochromatic light with a 4.5 mm pupil diameter.

For the AT LARA 829MP lens, the TF-MTF demonstrated a low-addition bifocal profile, with the best optical performance at the far focus (Figure 2e,f). These findings agree with those reported previously [8,30,31]. However, in the evaluation using blue and red wavelengths (Figure 3c,d), different patterns were observed for each wavelength. These results are consistent with those obtained by Łabuz et al. [7], with the proviso that the axial shift in the red and blue MTFa curves was induced by the spherically aberrated cornea used in their optical setup. Interestingly, the results obtained with the AT LARA 829MP lens are comparable to those reported by Millán and Vega [32] for the Symfony IOL (Johnson & Johnson, Santa Ana, CA, USA), indicating that both lenses share a similar optical design. Regarding halo formation (Figure 4), no previous studies evaluating halo formation at the main foci of this lens were found in the literature. Our results qualitatively show that the halo effect was lower at the far focus compared with the intermediate focus.

For the AcrySof IQ Vivity lens (Figure 2g,h), as the pupil diameter increased, optical performance improved at the distance focus but decreased across the rest of the vergence range. These TF-MTF results are consistent with those previously reported by other researchers [33,34]. Monochromatic MTFa curves (λ = 550 nm) exhibit EDoF behavior for both pupil conditions, with enhanced performance at intermediate distances for a 3.0 mm pupil (Figure 3g,h). These findings are in agreement with previous studies [33,34,35]. With regard to chromatic performance, no studies evaluating this lens across different wavelengths were identified. Regarding the haloes (Figure 4), this lens exhibited the smallest halo extent among the four MIOLs evaluated, at both vergences.

It is important to consider that the lens material also influences the chromatic behavior of intraocular lenses [36]. In general, the higher the Abbe number (and thus the lower the refractive index), the lower the chromatic dispersion and, consequently, the LCA. In this study, this effect may be observed when comparing the LCA at the far focus (diffraction order *m* = 0) of the FineVision lens (n = 1.46) with that of the AcrySof IQ Vivity lens (n = 1.55). However, it should be noted that the diffractive profile of each lens also has a direct effect on the magnitude of the LCA, as previously discussed.

This study is subject to certain limitations inherent to in vitro optical bench testing. First, MIOLs with different base powers were evaluated. Although, as previously mentioned, the Badal system substantially mitigates the impact of power-related magnification differences in our optical bench setup, such effects cannot be entirely excluded. Second, as only one sample per MIOL model was assessed, minor manufacturing variations may have influenced the results. Finally, the ISO 11979-2:2014 Model 1 cornea was employed, which does not reproduce the inherent SA of the human cornea. Nevertheless, this model was selected with the objective of isolating the effect of chromatic aberration from that of SA in the defocus curves [9], since both can induce an extension of focus depending on the SA of the lens [13]. In fact, if only the spherical aberration of models 1 and 2 were considered, it would be reasonable to assume that, with model 1, the results for MIOLs with SA = 0 would be better than those obtained with Model 2. Conversely, for lenses with SA < 0, the cornea Model 2 would yield better results, since the MIOL would at least partially compensate for the corneal SA. In this context, it is worth noting that the Acriva Trinova lens was also evaluated by Labuz et al. [13] using a commercial device with polychromatic illumination and both corneal Models. As their results with Model 1 for 3.0 mm and 4.5 mm pupils are consistent with ours, it is reasonable to extrapolate predictions for other lens models, particularly for the FineVision lens, given that it exhibits the same SA as the Acriva Trinova. The differences reported in that study indicate that, in general, higher lens SA results in smaller discrepancies between outcomes obtained with both corneal models and for condition 2, there is a decrease in the distance MTFa, a smoothing of the intermediate and far foci, and a shift in the near focus.

Therefore, future research should consider evaluating these lenses using corneal models that incorporate physiological aberrations, in order to enhance the clinical relevance and extrapolation of optical bench findings.

## 5. Conclusions

In summary, this study presents a comprehensive in vitro evaluation of the optical performance of four premium MIOLs. Each lens demonstrated distinct optical performance determined by its specific optical design. As prescribed by the ISO 11979 standard, design-related differences are evident inspecting the monochromatic TF-MTFs. Nevertheless, we confirmed that this metric is insufficient to fully characterize a MIOL, as it does not capture relevant information related to polychromatic response and possible halo formation. This is especially true for EDoF designs.

Our results confirmed that, apodized trifocal lenses exhibited different behaviors due to the different diffraction orders intended to generate the different foci. In addition, the FineVision lens demonstrated greater pupil dependence than the Acriva Trinova, favoring distance vision under mesopic pupil conditions. A similar behavior was observed in the EDoF lenses.

With respect to halo formation, noticeable differences were identified among the four MIOLs. EDoF designs produced fewer halos than trifocals. Specifically, the AcrySof IQ Vivity lens showed the smallest halo extent, while the FineVision POD F lens generated dimmer but more extended halos at the far focus.

The experimental TF-MTF and MTFa findings were consistent with previously reported results. Minor discrepancies, likely attributable to variations in measurement setups or methodologies, occasionally hinder direct comparison; however, in this study, the use of the same experimental setup for all four lenses enables direct and reliable comparison between them.

In conclusion, by addressing existing gaps in the literature concerning in vitro evaluation of these four MIOL models, the present work provides clinically relevant insights to guide ophthalmologists in selecting the most appropriate lens according to individual patient visual demands.

## Figures and Tables

**Table 1:** Main specifications of the multifocal intraocular lens (MIOLs) included in this study.

	Fine Vision POD F	Acriva Trinova Pro C	AT LARA 829MP	AcrySof IQ Vivity
Optical design	Diffractive, aspheric	Diffractive, aspheric	Diffractive, aspheric	“X-Wave™”, aspheric
Power (D)	13	20	14	20
Material	Hydrophilic (26%) acrylic	Hydrophilic	Hydrophilic (25%) acrylic	Hydrophobic Acry-late/Methacrylate Copolymer
Refractive index	1.46	1.46	1.46	1.55
Abbe number	58	58	56.5	37
SA (μm)	−0.11	−0.10	0.00	−0.20
Addition’s diffraction Orders	Far: *m* = 0; Inter.: *m* = +1 Near: *m*′ = +1	Far: *m* = −1; Inter.: *m* = 0; Near: *m* = +1	Far: *m* = +1; Inter.: *m* = +2;	-

**Table 2:** Experimental Through-Focus Modulation Transfer Function (TF-MTF) values at the main foci obtained for each lens evaluated for the spatial frequency of 50 lp/mm and for the pupil apertures of 3.0 mm and 4.5 mm.

3.0 mm	Far	Intermediate	Near
Fine Vision POD F	0.37	0.16	0.31
Acriva Trinova Pro	0.46	0.23	0.32
AT LARA 829MP	0.38	0.33	-
AcrySof IQ Vivity	0.37	0.13	-
4.5 mm			
Fine Vision POD F	0.41	0.12	0.22
Acriva Trinova Pro	0.43	0.27	0.30
AT LARA 829MP	0.44	0.25	-
AcrySof IQ Vivity	0.43	0.12	-

**Table 3:** Experimental area under the Modulation Transfer Function (MTFa) values at the main foci for each lens for the spatial frequency range of 0 lp/mm to 50 lp/mm, at λ = 550 nm, with pupil apertures of 3.0 and 4.5 mm.

3.0 mm	Far	Intermediate	Near
Fine Vision POD F	23.9	12.2	17.0
Acriva Trinova Pro	26.1	16.7	19.1
AT LARA 829MP	24.9	20.6	-
AcrySof IQ Vivity	29.3	22.8	-
4.5 mm			
Fine Vision POD F	26.1	7.0	12.1
Acriva Trinova Pro	25.5	18.5	18.0
AT LARA 829MP	28.5	15.4	-
AcrySof IQ Vivity	35.0	14.9	-

**Table 4:** Experimental longitudinal chromatic aberration (LCA) values at the main foci of each evaluated lens, with pupil apertures of 3.0 mm and 4.5 mm.

3.0 mm	Far	Intermediate	Near
Fine Vision POD F	+0.3 D	−0.2 D	−1.0 D
Acriva Trinova Pro	+0.9 D	+0.2 D	−0.3 D
AT LARA 829MP	−0.4 D	−1.1 D	-
AcrySof IQ Vivity	+0.7 D	-	-
4.5 mm			
Fine Vision POD F	+0.2 D	−0.4 D	−1.1 D
Acriva Trinova Pro	−0.9 D	+0.2 D	−0.4 D
AT LARA 829MP	−0.4 D	−1.2 D	-
AcrySof IQ Vivity	+0.8 D	+1.2 D	-

**Table 5:** Experimental peak intensity values of pinhole images measured at the main focus of each lens (λ = 550 nm, pupil diameter = 4.5 mm). Intensities are expressed on a logarithmic scale.

	Far	log_10_ Intermediate	Near
Fine Vision POD F	−0.20	−0.56	−0.38
Acriva Trinova Pro	−0.18	−0.19	−0.19
AT LARA 829MP	−0.19	−0.21	-
AcrySof IQ Vivity	−0.19	−0.22	-

## Data Availability

Dataset available on request from the authors.
